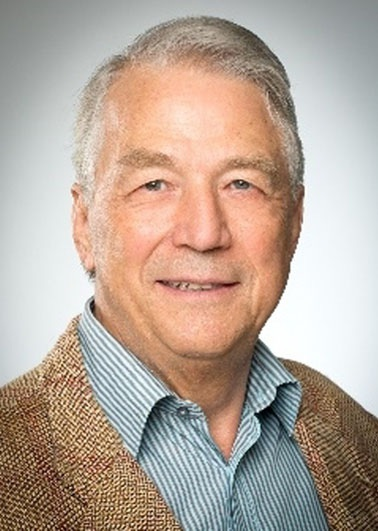# Antivitamins B_12_—Some Inaugural Milestones

**DOI:** 10.1002/chem.202003788

**Published:** 2020-11-03

**Authors:** Bernhard Kräutler

**Affiliations:** ^1^ Institute of Organic Chemistry and Center for Molecular Biosciences (CMBI) University of Innsbruck 6020 Innsbruck Austria

**Keywords:** antibiotic, cobalamin, growth inhibitor, transition metal, vitamin

## Abstract

The recently delineated structure‐ and reactivity‐based concept of antivitamins B_12_ has begun to bear fruit by the generation, and study, of a range of such B_12_‐dummies, either vitamin B_12_‐derived, or transition metal analogues that also represent potential antivitamins B_12_ or specific B_12_‐antimetabolites. As reviewed here, this has opened up new research avenues in organometallic B_12_‐chemistry and bioinorganic coordination chemistry. Exploratory studies with antivitamins B_12_ have, furthermore, revealed some of their potential, as pharmacologically interesting compounds, for inducing B_12_‐deficiency in a range of organisms, from hospital resistant bacteria to laboratory mice. The derived capacity of antivitamins B_12_ to induce functional B_12_‐deficiency in mammalian cells and organs also suggest their valuable potential as growth inhibitors of cancerous human and animal cells.

## Graphical abstract

**Like a wolf in a sheep's clothing** antivitamins B_12_ resemble vitamin B_12_ at the outside, but differ by the chemical character of their inside. As reviewed here, such B_12_‐mimics with a specifically designed metal‐centre are now available, having the potential of inducing devastating B_12_‐deficiency in B_12_‐dependent organisms, from mammals to microorganisms. Their application may provide biological and biomedical insights that induce the discovery of relevant applications in pharmacology and medicine.

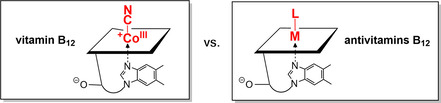

## Introduction

Vitamin B_12_, the Co^III^‐corrin cyanocobalamin (CNCbl), is a most fascinating and intriguing natural product,^[1]^ that was discovered as the original isolation form of the life‐saving ‘extrinsic’ anti‐pernicious anemia factor.^[2]^ An exceptional 5,6‐dimethyl‐benzimidazole pseudonucleotide appendage to the corrin core coordinates to the cobalt‐centre of CNCbl, establishing the unique and characteristic three‐dimensional architecture of the cobalamins (Cbls). Cbls belong to the larger family of the cobamides (Cbas), also including the related natural ‘complete’ corrinoids^[3]^ with other pseudonucleotide heterocycles[^3a^, ^4^] or linker units.^[5]^ These complex cobalt‐corrins are all generated in Nature by intricate B_12_‐biosynthetic paths,^[6]^ an exclusive capacity of some bacterial procaryotes and archaea.^[6b]^ Indeed, according to Eschenmoser's proposal, the natural B_12_‐derivatives may originate from structurally simpler cobalt‐corrinoid precursors, presumed to have developed in early forms of life.^[7]^

**Figure 1:**
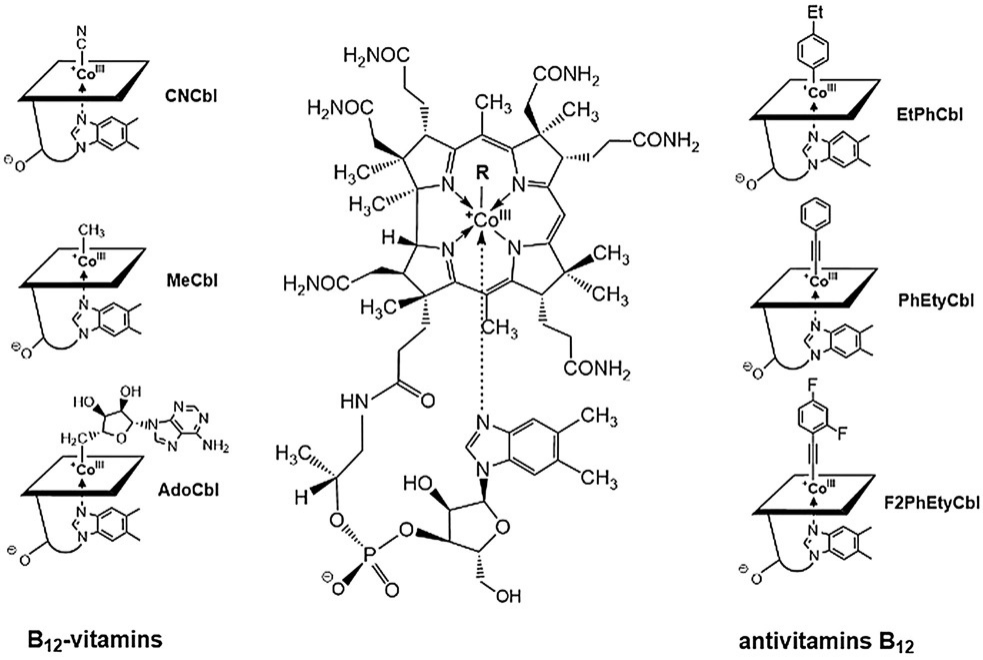
General structural formula of the cobalamins (centre), symbolic formulae of some important B_12_‐vitamers (left: vitamin B_12_ (CNCbl), methylcobalamin (MeCbl) and coenzyme B_12_ (AdoCbl)), and of (potential) Cbl‐based antivitamins B_12_ (right: the aryl‐Cbl EtPhCbl and the alkynyl‐Cbls PhEtyCbl and F2PhEtyCbl).

In spite of many years of intense medicinal,^[8]^ molecular biological and biochemical^[9]^ research, new physiological roles of B_12_ in humans keep emerging,[^8e^, ^10^] while some further Cbl‐related medical findings remain puzzling,^[11]^ so that B_12_ has been classified as a ‘moonlighting’ vitamin.^[12]^ The association of the B_12_’s own cobalt with a ‘Kobold’, the German word for goblin, appears to fit the occasionally puzzling situation. In fact, vitamin B_12_ (CNCbl) itself is not a directly physiologically active vitamin in humans and other mammals.[^8e^, ^13^] In order to set free its functional capacity, CNCbl needs to be converted by the mammalian metabolism,[^10c^, ^13^] into the organometallic B_12_‐cofactors methylcobalamin (MeCbl) and coenzyme B_12_ (adenosylcobalamin, AdoCbl).^[3c]^ CNCbl has, thus, the role of a ‘provitamin’.^[14]^ In fact, various Cbls, more directly functional physiologically than CNCbl, among them AdoCbl, are preferred B_12_‐vitamers for the treatment of some patients (Figure 1).^[15]^

The possible physiological effects of artificial intact Cbls designed to closely mimic the molecular shape of vitamin B_12_ and to resist metabolic conversion into the B_12_‐cofactors, have begun to attract our interest.^[16]^ The highly efficient and complex B_12_‐uptake and transport system in humans^[17]^ and higher animals^[18]^ should bind such inactive vitamin B_12_ analogues rather indiscriminately (as would, typically, also be the case for B_12_‐using bacteria^[19]^), with the consequence of the cellular import of (inactive) B_12_‐dummies competing with the natural cobalamins and effectively impairing B_12_‐metabolism. In consequence, B_12_‐analogues designed according to these criteria, would act as antivitamins B_12_ that induce functional Cbl‐deficiency in humans and other mammals in vivo; a concept presented in this Journal about 5 years ago.^[16]^ Antivitamins B_12_ relate to the broader class of the B_12_‐antimetabolites and were discussed in this context.[^14^, ^20^] Typical B_12_‐based antimetabolites, which are not covered in this Minireview, are Cbls (or other Cbas), modified at their periphery, that counteract, or fail to fulfil adequately, the physiological roles of natural B_12_‐derivatives in various B_12_‐dependent organisms, including many microorganisms. B_12_‐deficiency deprives some bacteria, animal and human cells of vital metabolic processes, which is a desirable consequence of the administration of metabolism based antibiotics and anti‐cancer agents.[^14a^, ^16^, ^20a^] Hence, broad biological^[3]^ and biomedical research interests[^8^, ^12^, ^14^, ^16^, ^20^, ^21^] are exploring means of inducing (functional) B_12_‐deficiency and are devoted to studies of its pathological effects.^[8]^

### From vitamin B_12_ to antivitamins B_12_—the cobalamin strategy

In line with the original concept,^[16]^ the complete Cbl‐scaffold of vitamin B_12_ was used as starting point for a (most efficient) preparation of antivitamins B_12_. The aryl‐Cbl 4‐ethylphenyl‐cobalamin (EtPhCbl), a novel type of organometallic B_12_‐derivative, was generated as a first such Cbl‐based antivitamin B_12_ (Figure 1).^[22]^ The critical design criteria for EtPhCbl were (i) its predicted (and verified) structural similarity with for example, CNCbl and (ii) its expected resistance against the metabolic removal of its aromatic capping group by the cellular ‘B_12_‐tailoring’ enzyme CblC,^[10c]^ thus inhibiting a later conversion into the organometallic B_12_‐cofactors.[^16^, ^22^] The aryl‐Cbl EtPhCbl bound well to the human B_12_‐transporter proteins, intrinsic factor, transcobalamin and holocobalamin, and was resistant against its tailoring by the enzyme CblC, as postulated.^[22]^ Most critically, EtPhCbl also led to functional Cbl‐deficiency in experiments with laboratory mice.^[23]^ However, while fulfilling the criteria of an antivitamin B_12_, EtPhCbl is photosensitive and visible light degrades it into the B_12_‐vitamer hydroxocobalamin (HOCbl),^[22]^ although with a low quantum yield.^[24]^ Hence, since EtPhCbl has the (often undesirable) property of a ‘photo‐conditional antivitamin B_12_’,^[24]^ our interest has turned to light stable Cbl‐based B_12_‐dummies. Suitable variants of the barely explored alkynylcobalamins^[25]^ with a strong organometallic Co−C_sp_ bond appeared attractive as presumed light stable potential antivitamins B_12_.^[26]^ The previously unknown phenylethynyl‐cobalamin (PhEtyCbl) was prepared, which turned out to be slightly hydrolysis‐sensitive, but was light stable and thermally robust and exhibited similar binding‐affinity as CNCbl for the human proteins of B_12_‐transport.^[26a]^ Furthermore, the fluorinated 2,4‐difluorophenyl‐derivative F2PhEtyCbl was not only light‐stable,^[27]^ but also rather inert against acid‐induced hydrolytic cleavage of its Co−C bond, as expected.^[26b]^ F2PhEtyCbl bound and inhibited the holoenzyme CblC loaded with the co‐substrate glutathione, allowing for a first crystal‐structure analysis of fully assembled human CblC.^[26b]^ Investigations, not only from our laboratory, but also from the Gryko group,^[28]^ have meanwhile expanded the methodology for the preparation of organometallic alkynyl‐cobalt‐corrinoids. Indeed, the robust alkynyl‐Cbls have become attractive potential cellular import vehicles (‚Trojan Horses’) with a range of biological and biomedical applications.[^28b^, ^29^]

### Engineered B_12_‐biosynthesis opens direct non‐cobalt synthesis‐paths to antivitamins B_12_

**Figure 2:**
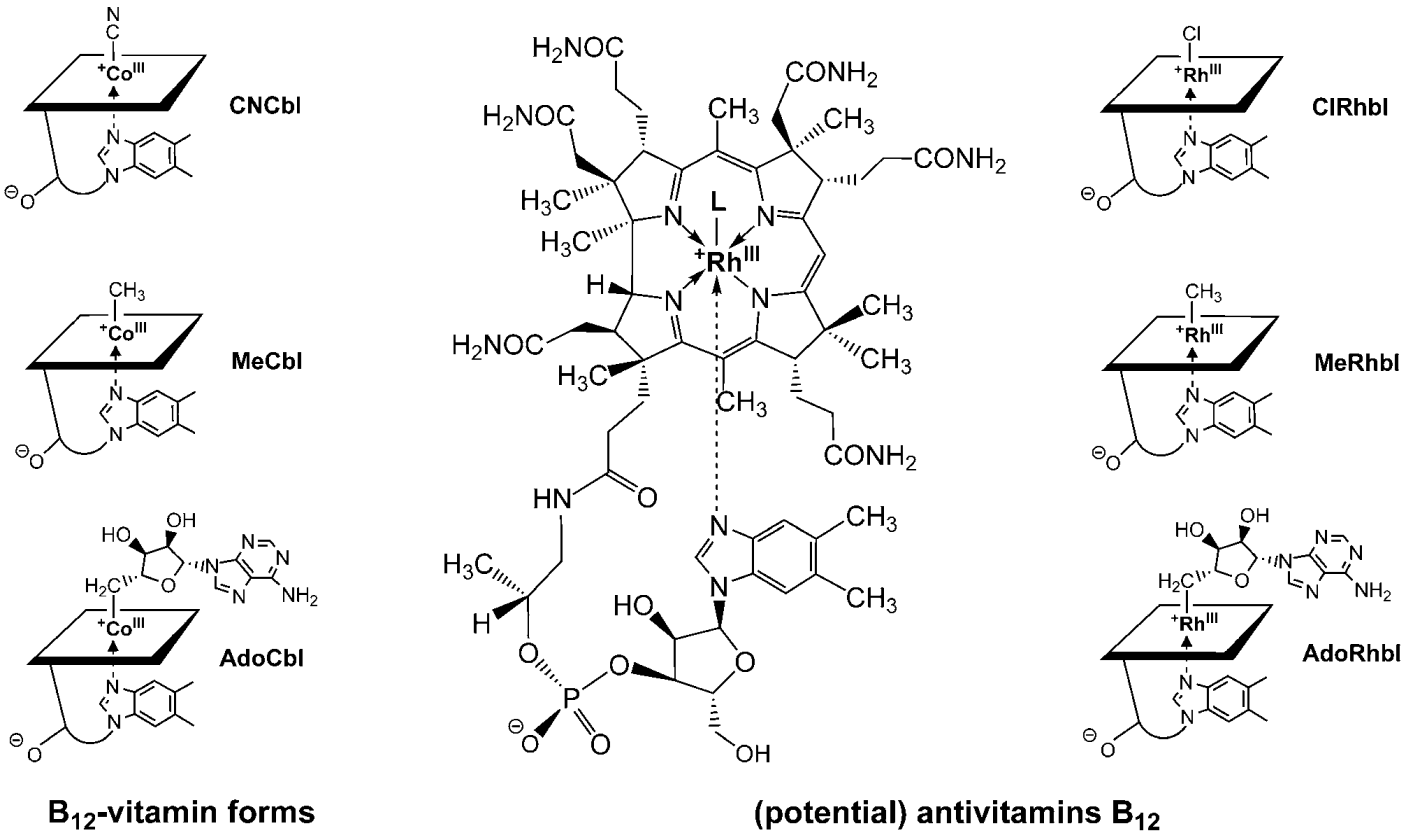
Left. Symbolic formulae of some important B_12_‐vitamers: vitamin B_12_ (CNCbl), methylcobalamin (MeCbl) and coenzyme B_12_ (AdoCbl); centre: General structural formula of the rhodibalamins; right: Symbolic formulae of three rhodibalamins as (potential) antivitamins B_12_: chloro‐Rhbl (ClRhbl), methyl‐Rhbl (MeRhbl) and adenosyl‐Rhbl (AdoRhbl).

The possible conversion of aryl‐ and alkynyl‐Cbls into the B_12_ vitamers hydroxocobalamin (HOCbl) or aquocobalamin (H_2_OCbl), by light or acid, respectively, was seen as a drawback as to their use as antivitamins B_12_,[^22^, ^26a^] prompting us to look out for strategic alternatives. Indeed, our simple structure‐based design criteria for the antivitamins B_12_, that is, structural similarity with CNCbl and resistance against metabolic tailoring by the enzyme CblC,^[16]^ would not only be an inbuilt feature of some inert Cbls, but a select and suitably designed group of metbalamins (Metbls),[^14b^, ^30^] transition metal analogues of the Cbls, might also serve this purpose. In this respect, rhodium, the group IX homologue of cobalt, appeared to offer a most promising access to effective potential antivitamins B_12_, by furnishing rhodibalamins (Rhbls), the Rh‐based Cbl‐analogues, presumed to be largely iso‐structural to corresponding Cbls.^[16]^ (Figure 2).

In the 1970s Koppenhagen and co‐workers reported the preparation of partially characterized Rhbls.^[30]^ In their exploratory tests with microorganisms and human cell cultures, adenosylrhodibalamin (AdoRhbl),^[31]^ the Rh‐homologue of AdoCbl, was indicated to behave as a B_12_ antimetabolite.^[32]^ We have recently developed an intricate chemical‐biological total synthesis of AdoRhbl in a team with the Warren group in Canterbury (UK). AdoRhbl was first synthesized using the biotechnologically prepared natural metal‐free B_12_‐ligand hydrogenobyrinic acid a,c‐diamide (Hbad)[^6b^, ^33^] as starting material, followed by an adequate cocktail of further chemical and enzymatic transformations of Hbad.^[34]^ AdoRhbl was fully characterized in detail as a close structural, non‐functional AdoCbl mimic that efficiently inhibited an AdoCbl‐dependent enzyme diol dehydratase, as well as the growth of the bacterial pathogen *Salmonella enterica*.^[34]^ In an additional welcome contrast to the antivitamin B_12_ EtPhCbl and to the coenzyme AdoCbl, the related AdoRhbl proved stable when irradiated with sunlight.[^31^, ^34^]

**Figure 3:**
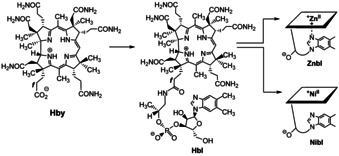
Biosynthetic hydrogenobyric acid (Hby) is starting material for the partial synthesis of hydrogenobalamin (Hbl), a direct synthesis platform for transition metal analogues of vitamin B_12_ (Metbls), such as zincobalamin (Znbl) and nibalamin (Nibl).

As Rhbls, the Rh‐analogues of the Cbls, appeared to constitute a group of promising antivitamins B_12_, a systematic and more direct synthesis methodology of Rhbls was developed. Its basis was a newly bioengineered preparative route to the now thoroughly characterized metal‐free B_12_‐ligand hydrogenobyric acid (Hby).^[35]^ The metal‐free Hby also constituted an excellent basis for the partial synthesis of hydrogenobalamin (Hbl), the complete metal‐free ligand of the Cbls (Figure 3).^[36]^ The metal‐free Hbl, in turn, is a rational general starting material for the synthesis of specific Metbls, a long‐standing dream and topical subject in the B_12_‐field,[^14b^, ^30^, ^37^] and in bioinorganic chemistry.^[38]^ The biosynthetically availabile Hbl has meanwhile served in our hands for the one‐step synthesis of chlororhodibalamin (ClRhbl),^[39]^ and from there, of methylrhodibalamin (MeRhbl),[^31^, ^39^] that is, of the Rh‐analogues of chlorocobalamin (ClCbl)^[40]^ and of MeCbl,^[41]^ respectively (see Figure 2). As revealed by the crystal structures of the organometallic AdoRhbl^[34]^ and of the ‘inorganic’ ClRhbl^[39]^ Rh^III^‐corrins and Co^III^‐corrins are closely isostructural and the slightly larger Rh^III^‐ion appears to fit strikingly better into the corrin ligand of the Cbls than the ‘natural’ Co^III^‐ions.[^34^, ^39^]

The metal‐free B_12_‐ligands Hby and Hbl are starting materials, not only for the syntheses of Rhbls, but, obviously, also of other Metbls. So far, we have reported on the synthesis and on the detailed structural characterization of zincobalamin (Znbl), the Zn^II^‐analogue of vitamin B_12_,^[42]^ and of the novel Ni^II^‐analogue, nibalamin (Nibl)^[36]^ (see Figure 3). According to detailed structural and computational studies, the redox‐inactive penta‐coordinate (‘base‐on’) Znbl constitutes a luminescent structural mimic^[42]^ of the penta‐coordinate ‘base‐on’ Co^II^‐cobalamin (Cbl^II^).^[43]^ The tetra‐coordinate diamagnetic ‘base‐off’ Ni^II^‐corrin Nibl represents a largely redox‐inactive structural mimic of the highly activated tetra‐coordinate ‘base‐off’ Co^II^‐ and Co^I^‐Cbls.[^36^, ^45^] The reduced Cbls represent the often cryptic high‐energy intermediates in many Cbl‐dependent enzymatic reactions,[^3c^, ^44^, ^45^] as well as in some essential B_12_‐biosynthetic organometallic transformation, for example, as catalysed by adenosyl transferases.^[46]^

**Figure 4:**
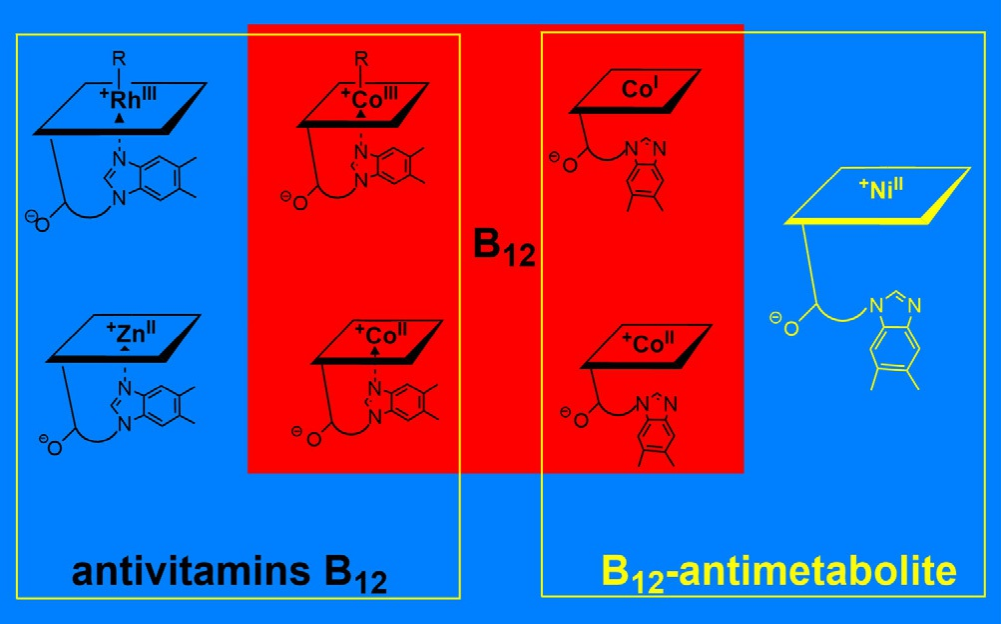
The Metbls Rhbls, Znbl and Nibl (blue field) are (largely) inert structural mimics of Co^III^‐, Co^II^‐ and Co^I^‐cobalamins (red background), and are efficient inhibitors of B_12_‐dependent enzymes useful for basic biochemical studies.

Together with the newly available hexa‐coordinate Rhbls, penta‐coordinate (‘base‐on’) Znbl and tetra‐coordinate (‘base‐off’) Nibl constitute a complete suite of structural transition metal mimics of the Cbls in their biologically accessible redox states, that is, hexa‐coordinate ‘base‐on’ Co^III^‐Cbls, penta‐coordinate ‘base‐on’ Co^II^‐ and tetra‐coordinate ‘base‐off’ Co^II^‐ or Co^I^‐Cbls, providing us with a structurally ‘complete’ small set of biochemically inactive B_12_‐antimetabolites, inhibitors of B_12_‐enzymes and (some of them) potential antivitamins B_12_ ^[36]^ (Figure 4). The ‘base‐on’ Metbls Rhbls and Znbl are likely to function as genuine antivitamins B_12_, the ‘base‐off’ Ni^II^‐analogue Nibl as a B_12_‐antimetabolite that inhibits some B_12_‐dependent enzymes but may not be bound well by the mammalian B_12_‐transporter proteins. Hence, in order to clarify the capacity of Metbls to serve as antivitamins B_12_ according to our concept,^[16]^ their ability to mimic the Cbls with respect to high‐affinity binding to the very structure‐selective B_12_‐uptake and transport system of humans (and other mammals) needs to be analysed.

### Application of antivitamins B_12_ induces functional B_12_‐deficiency

As delineated above, antivitamins B_12_ are structural Cbl‐mimics designed to counteract the effect of CNCbl (and of its B_12_ vitamer forms) in humans and animals by causing (functional) B_12_‐deficiency upon their cellular uptake,^[16]^ a deadly metabolic defect. Such an uptake of antivitamins B_12_ leads, first of all, to the inactivity of the mammalian B_12_‐dependent enzymes methionine synthase (MetH)^[47]^ and methylmalonyl‐CoA‐mutase (MCM)[^44^, ^48^] due to functional B_12_‐deficiency, detectable in the accumulation of homocysteine and methylmalonic acid, two biomarkers of B_12_‐deficiency.[^10d^, ^49^] Functional B_12_‐deficiency, induced by antivitamins B_12_ in humans and in other mammals, results, on the one hand, from the inability of these B_12_‐dummies to assume the specific ‘canonical’ roles of the B_12_‐cofactors of MetH and MCM, which are based on the organometallic reactivity of MeCbl and of AdoCbl, respectively.[^3c^, ^44^, ^45^] However, antivitamins B_12_ will, on the other hand, extensively mimic the (merely) structure‐based (‘non‐canonical’) regulatory functions of the Cbls, giving fake signals for the availability of genuine B_12_‐cofactors by imitating effectively their binding capacity to natural bio‐macromolecular targets, such as B_12_‐responsive regulatory proteins and RNA.[^16^, ^34^] As described below, a multitude of gene‐regulatory roles of the natural B_12_‐cofactors have been discovered in microorganisms.^[50]^ However, so far, in humans only two such bio‐macromolecular binding interactions have been detected.[^10a^, ^51^] Further ‘non‐canonical’ roles of Cbls in humans and in other mammals are suggested, for example, by the observation of a cytokine and growth‐factor imbalance in the central nervous system in laboratory rats due to Cbl‐deficiency,[^8d^, ^12^] as well as of irregular melanocyte homeostasis induced by B_12_‐deficiency in human cell cultures.^[52]^ Antivitamins B_12_ may be particularly helpful in imitating and identifying such puzzling roles, as well as in discovering new ‘non‐canonical’ ones.

### Antivitamins B_12_ as molecular probes

A range of remarkable recent discoveries in the B_12_‐field has put Vitamin B_12_ in the spotlight again.^[53]^ Indeed, B_12_‐derivatives play essential roles as organometallic biocatalysts,^[45]^ not only in humans, animals, bacteria and archaea but, surprisingly, in a range of algae, as well.^[54]^ Some forms of bacterial photo‐regulation involve natural cobamides,^[55]^ as do critical steps of the biosynthesis of photosynthetic tetrapyrroles^[6b]^ and of other complex metabolites,^[56]^ including the anaerobic metabolism of hydrocarbons.^[56d]^ Mechanistic insights into the exceptional biochemistry of the involved B_12_‐dependent enzyme reactions or means of the B_12_‐based control of essential cellular processes are areas of continuous interest. Studies with antivitamins B_12_ and other structurally characterized Metbls may potentially contribute to this subject,^[36]^ relying on two key structure‐based factors: (i) By imitating the structures of the B_12_‐cofactors or of reactive intermediate B_12_‐species in the course of enzyme reactions, suitably structured (inactive) B_12_‐mimics have an excellent capacity to inhibit the corresponding enzymatic steps. Hence, for example, the Ni^II^‐analogue of the cryptic intermediate Co^I^‐form cob(I)alamin inhibits an AdoCbl‐generating Ado‐transferase in an in vitro study^[36]^ (see above for corresponding pertinent findings with the alkynyl‐Cbl F2PhEtyCbl^[26b]^ and with AdoRhbl^[34]^). (ii) By mimicking the structures of the B_12_‐type ligands in B_12_‐dependent regulatory functions in various organisms, antivitamins B_12_ are, on the other hand, presumed to simulate the availability of the corresponding physiologically active B_12_‐derivatives, for example, via B_12_‐riboswitches^[57]^ and in B_12_‐responsive regulatory proteins.[^51^, ^58^] The observed strong growth‐inhibition of *Salmonella enterica* by AdoRhbl was, hence, ascribed to its specific binding to the *BtuB* B_12_‐riboswitch as a structural AdoCbl‐mimic, inhibiting the expression of a B_12_‐uptake protein in this microorganism.^[34]^ Similar further in vitro and in vivo experiments with AdoRhbl and some Cbl‐based antivitamins B_12_ have recently been carried out,^[59]^ signifying the ability of structurally competent antivitamins B_12_ to simulate the presence of physiologically functional Cbls. Indeed, as long as the cellular and organismal import of antivitamins B_12_ and of other Metbls by the natural pathways would be feasible, as expected, their capacity for generating functional B_12_‐deficiency should also be maintained in vivo, even in living animals.^[23]^

### Antivitamins B_12_ as antibiotics and as cellular growth‐inhibitors for human and animals

Antivitamins B_12_[^16^, ^60^] and other B_12_‐antimetabolites[^14^, ^20^, ^61^] may function as B_12_‐dummies and act as inhibitors of B_12_‐dependent enzymes, impairing the growth and reproduction of bacteria and of other microorganisms. This early explored effect of modified vitamin B_12_‐derivatives as B_12_‐antimetabolites (see for example[^3a^, ^20^]) could recently be extended to the critical case of hospital‐resistant Gram‐negative bacteria, where the broad antibiotic activity of sulfonamides was boosted decisively by the addition of the antivitamin B_12_ EtPhCbl to the bactericidal sulfonamide cocktail.^[60]^ Addition of the antivitamin B_12_ was proposed to result in an effective methylfolate trap,^[60]^ by blocking the formation of free tetrahydrofolate by methionine synthase. In addition to their proposed role in impairing the biosynthetic formation and in reducing the cellular availability of the (active) B_12_‐cofactors,[^16^, ^22^, ^23^, ^60^] antivitamins B_12_ may also intercept the uptake of the essential B_12_‐derivatives by B_12_‐dependent microorganisms due to their B_12_‐mimetic regulatory activity as ligands of (for example) B_12_‐riboswitches.^[59]^ Indeed, the response of B_12_‐regulatory elements to binding of a B_12_‐type ligand is expected not to differentiate between the functional classification of the latter as ‘vitamin’ or as ‘antivitamin’. In consequence, both the ‘canonical’ bio‐catalytic and the ‘non‐canonical’ B_12_‐regulatory roles played by the natural cobamides bestow antivitamins B_12_ with a potentially very effective two‐pronged bactericidal activity, as verified recently with AdoRhbl, the rhodium analogue of AdoCbl.^[34]^

Since the deactivation of the B_12_‐dependent enzymatic processes in humans and other mammals leads to an impaired metabolism, disrupting physiological function[^8a^, ^21a^, ^62^] and also causing fundamental neuropathological deficiencies,^[63]^ regular cellular growth is inhibited as consequence of a (functional) B_12_‐deficiency. Antivitamins B_12_ may, hence, be useful as anti‐cancer agents.[^14b^, ^16^] As already explored in early in vitro investigations, B_12_ rhodium analogues were observed to inhibit as diversely active B_12_‐antimetabolites, the growth of human normo‐ and megalo‐blastic bone marrow cells.[^30^, ^32^] It will be of interest to learn more about the diagnostic and therapeutic applications of well‐characterized, pure antivitamins B_12_ as agents for anti‐cancer diagnosis and treatment in humans and other mammals. Indeed, suitably fluorescence labelled, radiolabelled and other bio‐conjugated B_12_‐derivatives have proved useful, over the recent years, as ‘Trojan Horses’ for the cellular import of diagnostic loads and for targeted drug delivery,[^20a^, ^64^] helpful in inhibiting the growth and the detection of malignant cells,[^64a^, ^65^] and useful for a range of other biomedical applications.^[66]^

## Summary and Outlook

Our original interest in the subject of antivitamins B_12_ was kindled by the expectation that these B_12_‐dummies would offer insights into functional B_12_‐deficiency in animals by an effective alternative methodology^[23]^ replacing total gastrectomy.^[67]^ This work has led to fruitful research collaborations, discovering new organometallic Cbl‐chemistry, photochemistry and biochemistry.[^22^, ^24^, ^68^] It has, likewise, opened up new avenues in the field of the fascinating transition metal analogues of the Cbls and of other natural corrinoids.[^34^, ^36^, ^39^, ^42^] The helical, ring‐contracted natural corrin ligand has been characterized as an exceptional ‘Procrustean Bed’ for bound transition metal ions, important for tightly binding and specifically activating the bound cobalt‐ions in their low‐spin states.^[35]^ As discovered with synthetic Ni^II^‐corrins,^[69]^ the natural corrin ligand also imposes the diamagnetic low‐spin state on bound Ni^II^‐ions,^[36]^ contrasting with the situation in related porphyrin‐type Ni^II^‐corphinoids.[^7^, ^70^] Interestingly, the 5,6‐dihydroxy‐corrin variant of a ‘B_12_‐type’ Ni^II^‐complex, recently prepared and studied in the Zelder group, also features a low‐spin 4‐coordinate Ni^II^‐centre.^[37b]^

Cbl‐based antivitamins B_12_ promise to represent exceptional antibiotics,^[60]^ an important area to be developed further in view of the acute problem of hospital‐resistant bacteria. As some bacteria use preferentially cobamides (Cbas) other than Cbls,^[71]^ the eventual adaptation of the methodology for the synthesis of Cbl‐based antivitamins B_12_ to the generation of corresponding Cba‐forms is expected to enhance their selective bacterial import as antibiotics, while simultaneously reducing the likelihood of the undesired uptake in human cells by their B_12_‐transporters.[^17a^, ^72^] In ongoing collaborative studies, antivitamins B_12_ and some other metbalamins are used as specifically targeted B_12_‐antimetabolites, under investigation with respect to their capacity to serve as, for example, enzyme inhibitors, as ligands of regulatory proteins and of B_12_‐riboswitches, as antibiotics, and as potentially useful anti‐cancer agents. Having now set up some inaugural milestones, a broad further impact of studies on antivitamins B_12_ and (further) B_12_‐transition metal analogues in the bio‐structural, biological and biomedical fields can be foreseen.

## Conflict of interest

The author declares no conflict of interest.

## Biographical Information

*Bernhard Kräutler is Emeritus Professor of Organic Chemistry at the Faculty of Chemistry and Pharmacy of the University of Innsbruck. He studied chemistry at the ETH in Zürich where he obtained a PhD, working with Professor Albert Eschenmoser. In 1991 he was called to the University of Innsbruck as Professor of Organic Chemistry. His recent research, besides antivitamins B_12_, concerns the structural, synthetic and biological chemistry of vitamin B_12_, as well as tetrapyrrolic natural chlorophyll catabolites from higher plants, named phyllobilins*.